# CXCR5 engineered human and murine Tregs for targeted suppression in secondary and tertiary lymphoid organs

**DOI:** 10.3389/fimmu.2025.1513009

**Published:** 2025-07-01

**Authors:** Matteo Doglio, Jyoti Rana, Adriana Stucchi, Maite-Muñoz Melero, Alessia Ugolini, Tatiana Jofra, Cristiano Toma, Clara Bercher-Brayer, Pierluigi Carulli, Sandeep Kumar, Paolo Monti, Elisa Martini, Senthilkumar Thirumurugan, Moanaro Biswas, Chiara Bonini, Georgia Fousteri

**Affiliations:** ^1^ Experimental Hematology Unit, Division of Immunology Transplantation and Infectious Diseases (DITID), IRCCS San Raffaele Scientific Institute, Milan, Italy; ^2^ Herman B Wells Center for Pediatric Research, Indiana University, Indianapolis, IN, United States; ^3^ Diabetes Research Institute, Division of Immunology, Transplantation and Infectious Diseases (DITID), IRCCS San Raffaele Scientific Institute, Milan, Italy; ^4^ IRCCS San Raffaele Scientific Institute, Milan, Italy

**Keywords:** regulatory T cells (Tregs), C-X-C chemokine receptor type 5 (CXCR5), type 1 diabetes (T1D), pancreatic islet transplantation, hemophilia, chimeric antigen receptor (CAR), TCR fusion construct epsilon (TRuCε)

## Abstract

**Introduction:**

Secondary and tertiary lymphoid structures are a critical target of suppression in many autoimmune disorders, protein replacement therapies, and in transplantation. Although antigen-specific regulatory T cells (Tregs), such as chimeric antigen receptor (CAR) Tregs, generally persist longer and localize to target tissues more effectively than polyclonal Tregs in animal models, their numbers still progressively decline over time. A potential approach to maximize Treg activity in vivo is the expression of chemokine receptors such as CXCR5, which would enable localization of a greater number of engineered cells at sites of antigen presentation. Indeed, CXCR5 expression on follicular T helper cells and follicular Tregs enables migration toward lymph nodes, B cell zones, and tertiary lymphoid structures that appear in chronically inflamed non-lymphoid tissues.

**Methods:**

In this study, we generated human and murine CXCR5 co-expressing engineered receptor Tregs and tested them in preclinical mouse models of allo-immunity and hemophilia A, respectively. Additionally, we engineered a murine CXCR5 co-expressing clotting factor VIII (FVIII) specific T cell receptor fusion construct epsilon (FVIII TRuCe CXCR5) Treg to suppress anti-drug antibody development in a model of FVIII protein replacement therapy for hemophilia A.

**Results:**

In vitro, anti-HLA-A2 CXCR5+ CAR-Tregs showed enhanced migratory and antigen-specific suppressive capacities compared to untransduced Tregs. When injected into an NSG mouse model of HLA-A2+ pancreatic islet transplantation, anti-HLA-A2 CXCR5+ CAR-Tregs maintained a good safety profile allowing for long-term graft survival in contrast to anti-HLA-A2 CXCR5+ conventional CAR-T (Tconv) cells that eliminated the graft. Similarly, FVIII TRuCe CXCR5 Treg demonstrated increased in vivo persistence and suppressive capacity in a murine model of hemophilia A.

**Discussion:**

Collectively, our findings indicate that CXCR5 co-expression is safe and enhances in vivo localization and persistence in target tissues. This strategy can potentially promote targeted tolerance without the risk of off-target effects in multiple disease models.

## Introduction

Regulatory T cells (Tregs) play a cardinal role in the induction and maintenance of peripheral tolerance (1, 2). Quantitative and qualitative alterations in circulating Tregs have been documented in autoimmune diseases such as type 1 diabetes (T1D), characterized by reduced suppressive function, decreased stability of FoxP3 expression, and increased secretion of proinflammatory cytokines (IFN-γ, IL-17) (3, 4). Similarly, in the X-linked blood coagulation disorder hemophilia A, large mutations in the *F8* gene and lack of central tolerance can result in the mounting of inhibitory anti-drug antibody (ADA) responses to the recombinant FVIII protein, which is administered as replacement therapy (5).

A specific subset of Tregs called T follicular regulatory (Tfr) cells, express the transcription factor B cell lymphoma 6 (Bcl6), high levels of Programmed Death 1 (PD-1), and the C-X- C motif chemokine receptor 5 (CXCR5) (6). Tfr cells can modulate follicular helper T (Tfh) cell activation of B cells and possibly control undesired antibody development against FVIII in patients with hemophilia or donor-specific antibodies (DSA) in diabetic patients transplanted with pancreatic islets (7–9). Deficiencies in Tfr cells have been shown to promote humoral autoimmunity in mouse models and aberrant Tfh/Tfr ratios are found in several autoimmune conditions (10, 11), including the pancreatic lymph nodes (pLN) of organ donors with T1D (12), suggesting that enhancing Tfr cell function could contribute to disease remission and blunt undesired antibody responses.

There is substantial evidence supporting Treg homing to draining lymph nodes (LNs) and sites of inflammation to restore tissue-specific immune tolerance (13, 14). Requirements for localization to draining LNs versus peripheral tissues differ significantly, necessitating tailored Treg-based therapeutic strategies. Localization and retention of Tregs in LNs require the expression of specific chemokine receptors, such as C-C motif chemokine receptor 7 (CCR7) for the T-cell zone, CXCR5 for B cell follicles, and CD62L (15, 16). Signals guiding Treg migration and survival in peripheral tissues are often tissue specific. For instance, Tregs express gut-homing receptors such as α4β7 and C-C motif chemokine receptor 9 (CCR9) to modulate inflammatory bowel disease (IBD), whereas C-X-C motif chemokine receptor 3 (CXCR3), the chemokine receptor for CXCL10, is crucial for migration to Th1-driven inflammatory sites like the islets (13, 17, 18). In hemophilia, ADA responses are initiated in the spleen, where FVIII antigen presentation to Tfh cells depends on an orchestrated effort by several anatomically distinct antigen presenting cell subsets (19). DSA on the other hand, are initiated in the draining lymph nodes of the transplanted organ/tissue (20).

Tertiary lymphoid structures (TLS) are organized aggregates of immune cells forming in non-lymphoid inflamed tissues (21). Several cytokines and chemokines, along with their corresponding receptors, play roles in TLS formation, including lymphotoxins (LTs), tumor necrosis factor (TNF), CCL21, CCL19, and CXCL13 (22, 23). In non-obese diabetic (NOD) mice, TLS formation occurs in pancreatic islets and islet grafts, fueling the auto-/alloimmune response (24, 25). Interestingly, besides their role in limiting humoral responses in secondary lymphoid organs, Tfr cells appear to act at TLS, promoting the development of immunological memory and avoiding the expansion of autoreactive cells (26).

Tregs engineered to express a chimeric antigen receptor (CAR) or a T cell receptor fusion construct (TRuC) are promising therapeutic modalities to prevent and cure autoimmunity, promote transplant tolerance, or to prevent ADA and DSA formation (2, 27, 28). Anti-HLA-A2 CAR-Tregs have demonstrated efficacy in inhibiting xenogeneic graft-versus-host disease (GvHD) and promoting transplant tolerance more effectively than polyclonal Tregs, migrating to HLA-A2^+^ skin and islet grafts (29–31). Preclinical studies also showed that anti-HLA-A2 CAR-Tregs were more effective at lower doses compared to unmanipulated Tregs, potentially achieving more targeted suppression and leading to safer therapies with fewer off-target effects (32). Pierini et al. demonstrated that islet-specific CAR-Tregs prevented the rejection of the graft in a mouse model of pancreatic islet transplantation (33). These findings were further confirmed by Muller et al., who showed that anti-HLA-A2 CAR-Tregs prevented the rejection of HLA-A2^+^ pancreatic islets, preserving normal glucose control in a T1D preclinical model (29). Rana et al. demonstrated that FVIII TRuCε Tregs (which complexes scFv recognition to CD3ε) engaged the endogenous CD3-TCR complex, delivering controlled signaling and functional suppression of ADA responses to FVIII (27).

In this study, we tested a strategy to enhance the migratory ability of engineered Tregs toward secondary and tertiary lymphoid organs by co-expressing CXCR5. We developed human anti-HLA-A2 CAR-Tregs co-expressing CXCR5 using a bidirectional lentiviral (LV) system for pancreatic islet transplantation. Similarly, we engineered murine CXCR5 co-expressing FVIII TRuCε Tregs to suppress FVIII specific ADA responses in a murine model of hemophilia A (HA). *In vitro*, both human and murine CXCR5^+^ engineered Tregs showed superior migratory properties toward a CXCL13 gradient. Human CXCR5^+^ CAR-Tregs retained their polyclonal and antigen-specific suppressive abilities and, in contrast to CAR-T_convs_, anti-HLA CXCR5+ Tregs did not reject HLA-A2^+^ grafts in an NSG mouse model of pancreatic islet transplantation. In a HA mouse model, murine engineered Tregs displayed a preferential localization in secondary lymphoid organs and improved suppression of FVIII specific ADA response. Taken together, we demonstrate the benefit of CXCR5 co-transduction in enhancing the migratory properties of engineered Tregs into secondary and tertiary lymphoid organs without affecting their overall functional properties.

## Results

### Generation of anti-HLA-A2 CXCR5+ CAR-Tregs from human PBMC

Islet transplantation is a potential cure for type 1 diabetes (T1D), offering the possibility of insulin independence and improved glycemic control. However, the success of this therapeutic option is often limited by graft rejection mediated by both alloreactive and autoreactive T cells, as well as donor-specific humoral responses. In this context, the CXCR5-CXCL13 chemokine axis plays a significant role. CXCL13, produced by germinal center components, transplanted organs, and inflamed tissues recruits CXCR5^+^ B and T lymphocytes to these sites (34). This axis facilitates B-T cell interactions, promoting B cell maturation and antibody production in lymphoid organs.

**Figure 1:**
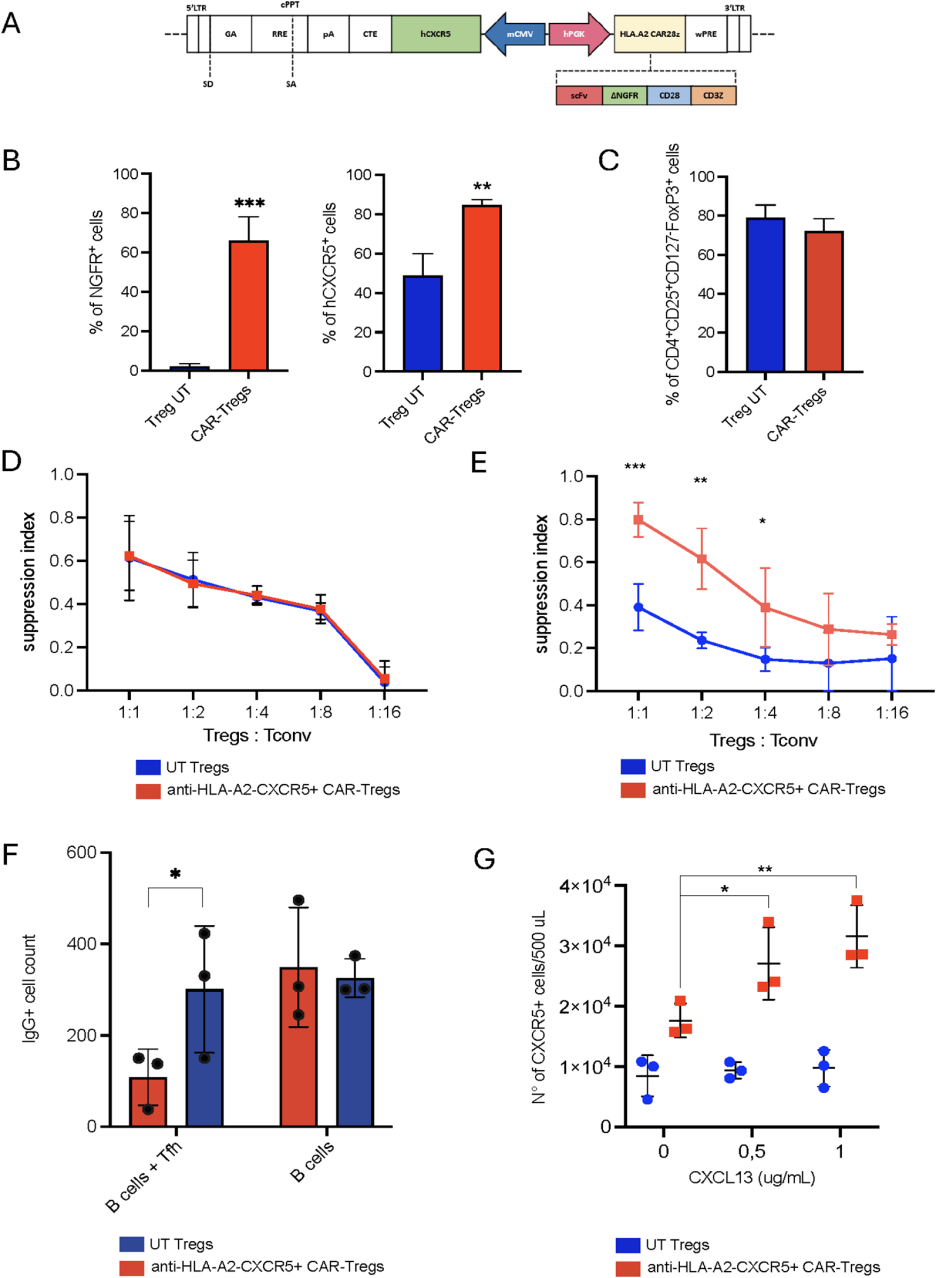
HLA-A2-CAR CXCR5^+^ Tregs *in vitro* generation and validation. **(A)** Schematic representation of the bidirectional lentiviral vector (LV). Anti-HLA-A2 Chimeric Antigen Receptor (CAR) in sense under the control of a human phosphoglycerate kinase (hPGK) promoter, composed by single chain fragment variant (scFv), a truncated form of the neuron growth factor receptor (NGFR) as a spacer, the transmembrane/intracellular human CD28 domain fused to the intracellular portion of the human CD3ζ chain. In anti-sense, the human C-X-C motif chemokine receptor 5 (CXCR5) gene under the control of a minimal Cytomegalovirus (mCMV) promoter. Other essential components necessary for functionality of the LV are indicated. LTR long terminal repeat, SD splice donor, SA splice acceptor, GA gag-pol element, RRE REV responsive element, cPPT central polypurine tract, pA polyadenylation signal, CTE constitutive transport element, WPRE woodchuck hepatitis virus post-transcriptional regulatory element. **(B)** Frequency of transduced cells, evaluated as percentage of CAR^+^ and hCXCR5^+^ Tregs by flow cytometry at Day +14. Cell transduction was assessed as percentage of NGFR^+^ cells. **(C)** Percentage of CD4^+^CD25^+^CD127^-^FoxP3^+^ cells in UT and CAR-Tregs at Day +14, assessed by flow cytometry. **(D)** *In vitro* polyclonal suppressive capacities and **(E)** antigen-specific suppressive capacities of UT or anti-HLA-A2 CXCR5^+^ CAR-Tregs. Results are expressed as Suppression Index, calculated as [1-(PBMCs’ proliferation with Tregs)/(PBMCs’ proliferation alone)] * 100. **(F)** *In vitro* B cell maturation assay in the presence of UT or anti-HLA-A2 CXCR5^+^ CAR-Tregs with or without T follicular helper cells (Tfh), expressed as the number of IgG^+^ B cells. **(G)** Migration of UT and anti-HLA-A2 CXCR5^+^ CAR-Tregs in response to varying concentrations of human CXCL13. Results are expressed as the number of migrating Tregs. For all the experiments reported in panel **(B-G)**, results are expressed as mean±SD (N = 3). 1 or 2-tailed Mann-Whitney test for **(B**, **F**, **G)** Two-way ANOVA with Tukey’s correction for **(E)** *p-value <0.05, **p-value < 0.01, ***p-value <0.001.

In organ transplantation settings, DSA produced in secondary and tertiary lymphoid organs can lead to tissue damage and graft rejection, as observed in pancreatic islet transplantation for T1D (35). To address these challenges, we engineered anti-HLA-A2 CXCR5^+^ CAR-Tregs from human peripheral blood mononuclear cells (PBMCs) to enhance their trafficking to lymphoid structures and improve their therapeutic efficacy in transplantation models. We first cloned the human *CXCR5* gene in antisense in a bi-directional lentiviral vector under the control of a minimal cytomegalovirus promoter (mCMV). Next, we employed a second-generation anti-HLA-A2 CAR, which was cloned in sense under the control of a human phosphoglycerate kinase (hPGK) promoter (**Figure 1A**). We isolated human CD4^+^CD25^+^ cells from healthy donor (HD) PBMCs, transduced them with the bi-directional lentiviral vector and expanded them in the presence of IL-2 and rapamycin, as previously described (28) (**Supplementary Figure 1A**). Anti-HLA-A2 CXCR5^+^ CAR-Tregs displayed high expression and co-localization of the two transgenes after 14 days of culture (respectively mean 66.10% ± 12.26% CAR expression and 84.80% ± 2.57% hCXCR5 expression) (**Figure 1B**, **Supplementary Figure 1B**) Engineered cells showed high purity in terms of Treg enrichment (mean Treg percentage 66.1% ± 12.3% for anti-HLA-A2 CXCR5^+^ CAR-Tregs and 84.8% ± 2.6% for UT Tregs) and an expansion rate comparable to untransduced (UT) Tregs (mean fold increase 34.1 ± 21.8 for CAR-Tregs and 32.2 ± 15 for UT Tregs) (**Figure 1C**, **Supplementary Figure 1C**). FoxP3^+^ CAR Tregs had >80% CD25 expression and >30% expression of Helios (considered a marker for Treg stability, **Supplementary Figures 1D, E**), and markedly higher MFIs of FoxP3, Helios, and CD25 expression (**Supplementary Figure 1F**) as compared to T_conv_ cells. Helios expression was markedly absent in T_convs_.

When functionally tested, anti-HLA-A2 CXCR5^+^ CAR-Tregs retained similar suppressive capacities as UT Tregs upon polyclonal stimulation (**Figure 1D**). Furthermore, anti-HLA-A2 CXCR5^+^ CAR-Tregs suppressed the proliferation of anti-NY-ESO-1 T_conv_ cells upon antigen-specific stimulation when cultured in the presence of HLA-A2^+^ NY-ESO-1^+^ feeder cells, thus confirming CAR functionality (mean of suppression index of 0.390 ± 0.108 for UT Tregs and 0.798 ± 0.079 for CAR-Tregs, p-value <0.05) (**Figure 1E**).

T follicular helper cells support B cell maturation and class-switch. To assess the effect on Tfh-B cell crosstalk, we isolated circulating HLA-A2^+^ B cells and CXCR5^+^ Tfh cells (**Supplementary Figure 1G**) and co-cultured them with either anti-HLA-A2 CXCR5^+^ CAR- or UT Tregs. Engineered cells significantly reduced the number of IgG^+^ B cells in the presence of Tfh compared to unmanipulated Tregs (IgG^+^ B cell number: 108.7 ± 61.5 in the CAR-Treg group vs 301.0 ± 138.8 in the UT group; p-value 0.03) (**Figure 1F**). To test their safety profile, engineered Tregs were co-cultured with an HLA-A2^+^ cell line. In contrast to anti-HLA-A2 CAR-T_convs_, anti-HLA-A2 CXCR5^+^ CAR Tregs exerted a much lower killing capacity toward the target (**Supplementary Figure 1H**).

To validate the functionality of the hCXCR5 transgene, we tested anti-HLA-A2 CXCR5^+^ CAR-Treg migratory capacity at different concentrations of CXCL13. Engineered Tregs showed superior migratory capacity compared to UT Tregs, directly proportional to the CXCL13 concentration (p-value <0.05 at CXCL13 0.5 μg/mL and p-value <0.01 at CXCL13 1 μg/mL) (**Figure 1G**). Collectively, these results demonstrate that anti-HLA-A2 CXCR5^+^ CAR-Tregs acquire superior migratory properties compared to UT Tregs *in vitro*, while retaining antigen-specific suppressive capacities against both T and B cells without eliminating their cognate target.

### *In vivo* safety and trafficking of human anti-HLA-A2 CXCR5^+^ CAR-Treg

**Figure 2:**
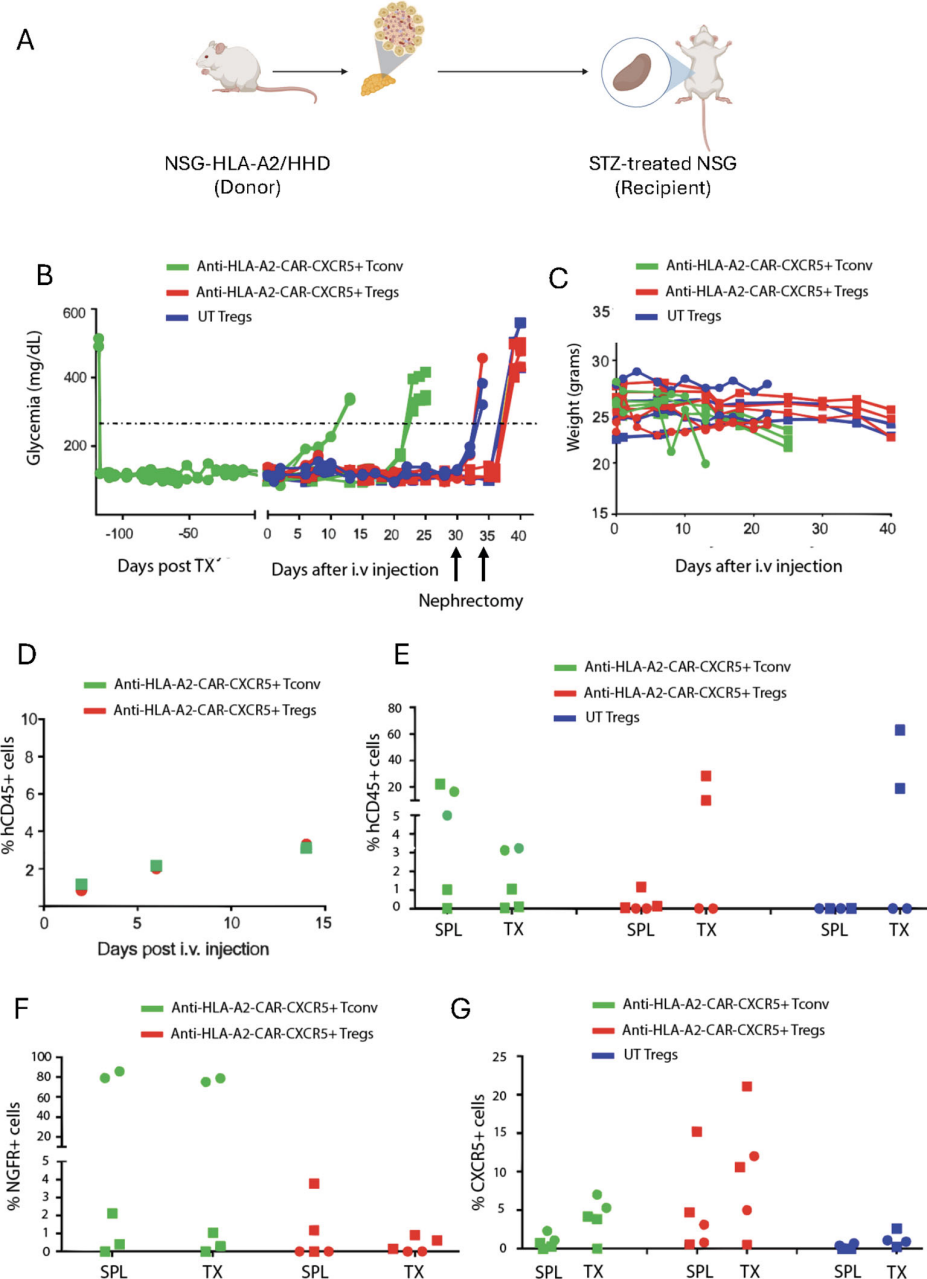
Anti-HLA-A2 CXCR5^+^ CAR-Tregs do not kill transplanted islets. **(A)** Experimental design. Five hundred HLA-A2^+^ pancreatic islets were isolated from HLA-A2 transgenic NSG mice and transplanted under the kidney capsule in HLA-A2^-^ NSG mice treated with streptozotocin to induce diabetes. Once glycemic control was achieved, mice were injected with 2x10^6^ of anti-HLA-A2 CXCR5^+^ CAR-T_conv_ or CAR-Tregs or UT Tregs. Glycemia was monitored in the blood and when reached values >250 mg/dl, the graft was considered rejected. Mice treated with Tregs underwent nephrectomy 30 days after the cell injection. **(B)** Blood glucose monitoring in transplanted mice assessed with a glucometer. TX = graft. **(C)** Weight monitoring in transplanted mice expressed in grams. **(D)** Percentage of circulating human CD45^+^ cells assessed by flow cytometry at different time points. **(E)** Frequency of human CD45^+^ cells in the spleen (SPL) and the graft (TX) at euthanasia, assessed by flow cytometry. Frequency of CAR^+^ **(F)** and CXCR5^+^ **(G)** cells in the spleen (SPL) and the graft (TX) at euthanasia, assessed by flow cytometry. For this experiment we employed a total of 5 animals in each group in two independent experiments, indicated by the square and the round symbols, respectively.

To evaluate the *in vivo* safety and trafficking of anti-HLA-A2 CXCR5^+^ CAR-Tregs, we used the kidney capsule islet transplantation model. NSG (HLA-A2 negative) streptozotocin (STZ)-induced diabetic animals were transplanted with pancreatic islets derived from HLA-A2 transgenic NSG mice under the kidney capsule (**Figure 2A**). After the establishment of normoglycemia, transplanted animals were infused with either anti-HLA-A2 CXCR5^+^ CAR-Tregs or CAR-T_conv_. To assess graft rejection, blood glucose was monitored every 5 days after cell injection (**Figure 2B**). UT Tregs served as a control group. Mouse weight was also recorded to monitor for signs of graft-versus-host disease (GvHD) (**Figure 2C**).

Islet rejection was observed in 5 out of 5 mice treated with anti-HLA-A2 CAR-T_conv_, within 25 days after treatment (**Figure 2B**). In contrast, none of the 5 mice that received anti-HLA-A2 CAR- or UT Tregs exhibited islet rejection. 30 to 35 days after cell infusion, the experiment was interrupted to confirm the functionality of the graft by removing the pancreatic islet bearing-kidney. All the mice returned to hyperglycemia few days after nephrectomy (**Figure 2B**).

Next, we assessed the frequency of circulating human leukocytes (hCD45^+^ cells) in the animals injected with engineered cells at different timepoints by flow cytometry, finding no differences (**Figure 2D**). At the end of the experiment, we evaluated the human infiltrate in the spleen and the transplanted pancreatic islets by flow cytometry. CAR-Treg treated mice showed a preferential accumulation of hCD45^+^ cells in the graft compared to the spleen, which were mainly composed by CXCR5^+^ CAR^-^ cells. Conversely, CAR-T_conv_ treated mice showed a similar frequency of hCD45^+^ and CXCR5^+^ cells between the graft and the spleen, with a high percentage of CAR^+^ cells in some animals (**Figures 2E–G**).

Altogether, these data demonstrated the good safety profile of anti-HLA-A2 CXCR5^+^ CAR-Tregs, which did not reject the transplanted pancreatic islets compared to CAR-T_convs_. In addition, the accumulation of engineered cells in the graft confirmed their migratory properties

### FVIII TRuCε CXCR5 Tregs are antigen specific

**Figure 3:**
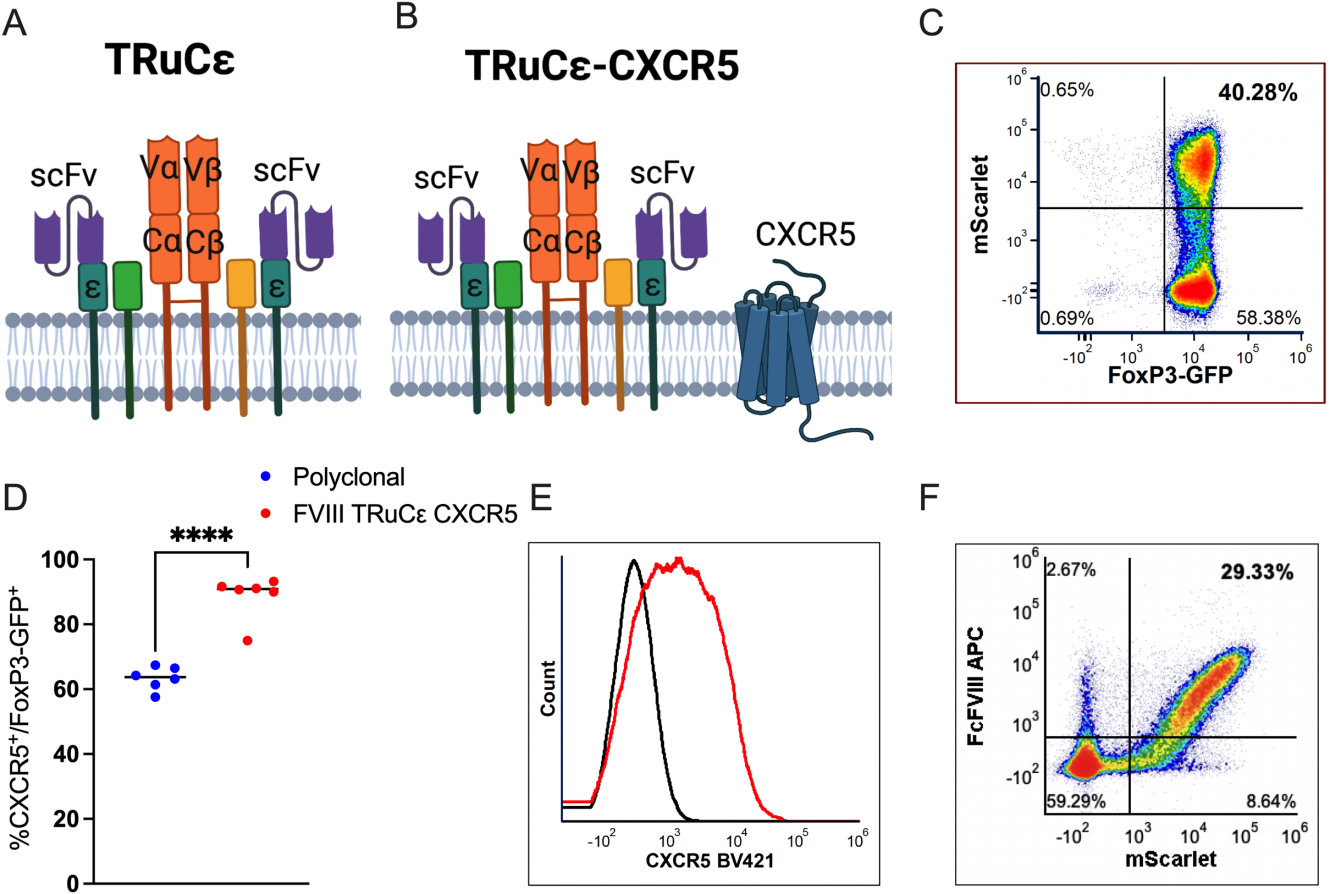
FVIII TRuCε CXCR5 Treg *ex vivo* generation and validation. **(A)** Surface organization of FVIII TRuCε. The FVIII specific scFv is fused to CD3ε by a linker. The synthetic construct can integrate into the endogenous Treg CD3-TCR complex. **(B)** FVIII TRuC**ε** CXCR5 co-expresses murine CXCR5 on the cell surface. **(C)** Representative density plot of FoxP3-GFP^+^ Tregs transduced with either **(A)** or **(B)** as indicated by mScarlet reporter protein expression. **(D)** Percentage of CXCR5^+^ expression in polyclonal and FVIII TRuCε CXCR5 transduced FoxP3-GFP^+^ Tregs. **(E)** Histogram overlay plots indicating overexpression of CXCR5 (red histogram) in FVIII TRuC**ε** CXCR5 transduced Tregs as compared to polyclonal Tregs (black histogram). **(F)** Representative density plot indicating binding of FcFVIII by mScarlet^+^ FVIII TRuC**ε** CXCR5 transduced Tregs as detected by APC conjugated α-IgG Fc secondary antibody. Data represents mean±SEM, ****p<0.0001 for **(D)** using unpaired t test.

Next, to generate the FVIII TRuCε synthetic receptor, an scFv with specificity to the immunogenic C2 domain of human FVIII (clone BO2C11) was synthesized, fused to murine CD3ε and cloned into a retroviral backbone that co-expressed the reporter protein mScarlet as described (27) (**Figure 3A**). The murine CXCR5 coding sequence was expressed downstream of the auto cleaving P2A peptide to generate the FVIII TRuCε CXCR5 receptor (**Figure 3B**). Transduced FoxP3-GFP Tregs maintained >98% GFP expression indicating no outgrowth by contaminating T_conv_ and ~40% Tregs were mScarlet^+^, indicating FVIII TRuCε expression following retroviral transduction and *ex vivo* expansion (**Figure 3C**). FVIII TRuCε CXCR5 transduction increased the frequency of CXCR5 expressing Tregs from 63.36±1.45% in polyclonal Tregs to 88.6±2.75% in engineered Tregs (**Figure 3D**). CXCR5 MFIs were also increased in engineered Tregs as compared to polyclonal Tregs (1044±206 vs 383±22, **Figure 3E**). FVIII TRuCε CXCR5 Tregs (mScarlet^+^) bound Fc-conjugated FVIII in a highly specific manner as detected by fluorochrome labeled anti-Fc antibody (**Figure 3F**).

### FVIII TRuCε CXCR5 Tregs respond to FVIII stimulation

**Figure 4:**
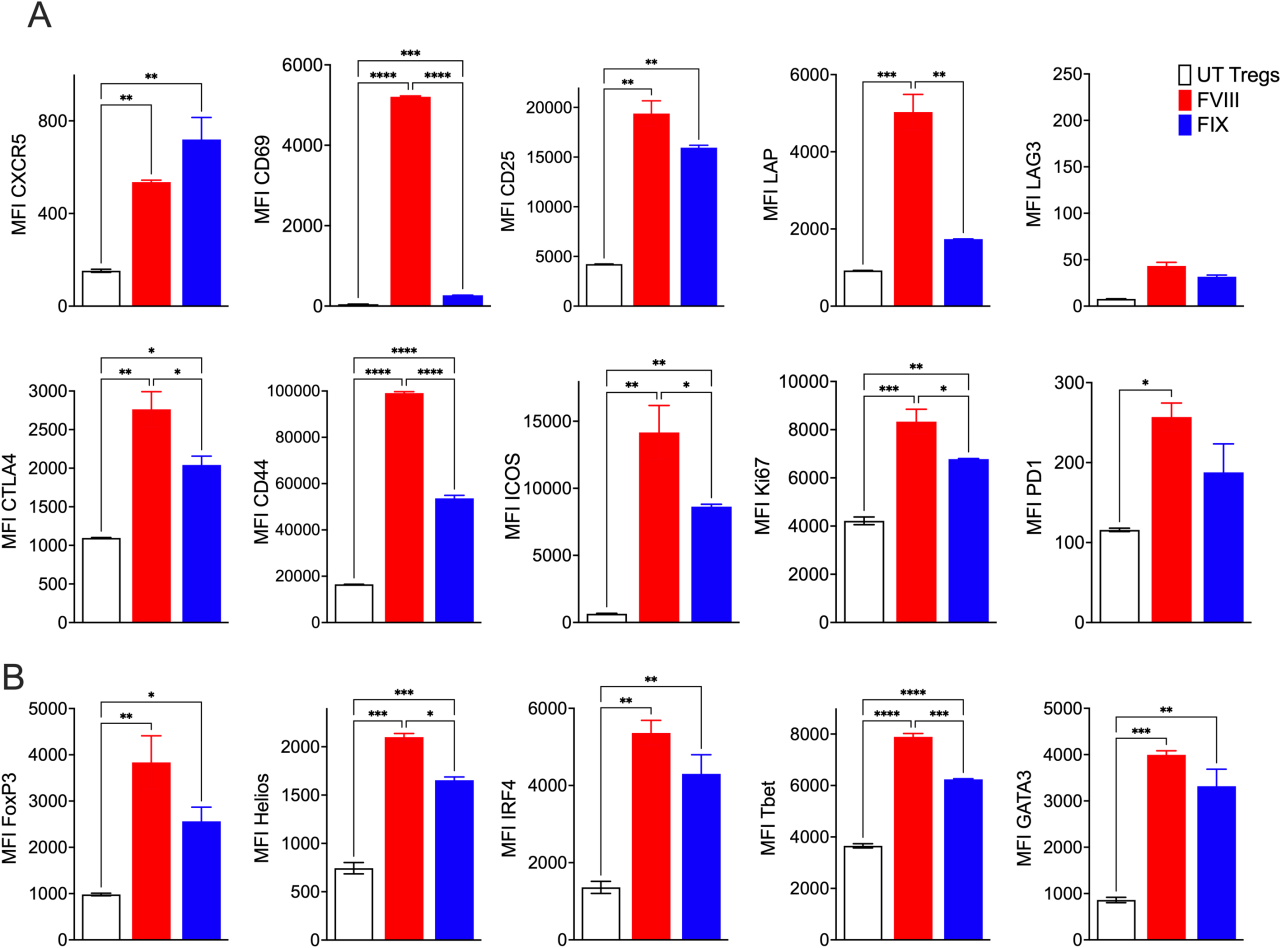
FVIII TRuCε CXCR5 Tregs respond to FVIII stimulation *in vitro.* **(A)** *In vitro* upregulation of activation markers CXCR5, CD69, CD25, LAP, LAG3, CTLA4, CD44, ICOS, Ki67 and PD1 in recombinant FVIII or FIX stimulated engineered FVIII TRuCε CXCR5 Tregs at 48hrs. **(B)** Upregulation of transcription factors FoxP3, Helios, IRF4, TBet and GATA3 in recombinant FVIII or FIX stimulated engineered FVIII TRuCε CXCR5 Tregs at 48hrs. Changes in MFI are quantified. Data represents mean±SEM, ^∗^p < 0.05, ^∗∗^p < 0.01, ***p<0.001, ****p<0.0001 using 1-way ANOVA with Sidak’s multiple comparisons analysis.

Flow cytometric phenotyping of FVIII TRuCε CXCR5 Tregs at 48h following FVIII stimulation showed significant antigen specific upregulation of canonical Treg activation markers such as CD69, CD25, LAP, CTLA-4, CD44, ICOS, Ki67, and PD1 as compared to freshly isolated polyclonal UT FoxP3-GFP Tregs or stimulation with an irrelevant antigen, factor IX (FIX) (**Figure 4A**). Interestingly, we noted lower expression levels of CXCR5 despite constitutive overexpression in FVIII stimulated TRuCε CXCR5 Tregs as compared to FIX stimulation (**Figure 4A**). This was likely due to internalization of CXCR5 upon activation as has been previously reported (36). Staining for transcription factors confirmed acquisition of an activated phenotype upon FVIII stimulation, with upregulation of IRF4, TBet, and GATA3 (**Figure 4B**). Both FoxP3 and Helios were increased in FVIII stimulated TRuCε CXCR5 Tregs (**Figure 4B**), indicative of an activated, effector Treg like phenotype (37–39).

### FVIII TRuCε CXCR5 Tregs show improved migration and persistence

**Figure 5:**
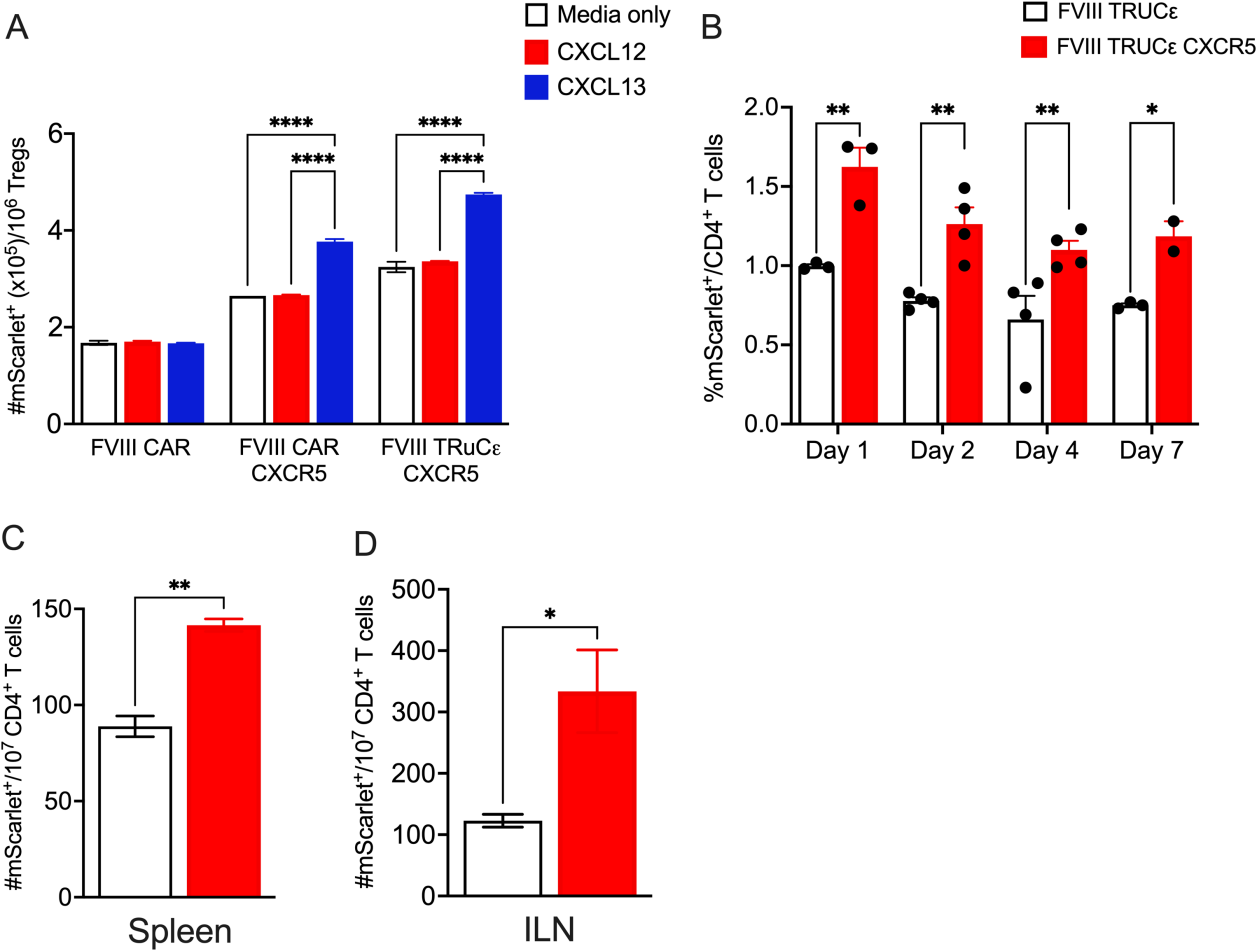
FVIII TRuCε CXCR5 Tregs display improved *in vitro* and *in vivo* persistence. **(A)** *In vitro* migration of FVIII CAR, FVIII CAR CXCR5, and FVIII TRuCε transduced Tregs through a transwell in response to either serum free media, CXCL12 or CXCL13 gradients. Number of migrated mScarlet^+^ cells at the bottom of the transwell are quantified by flow cytometry following 6hrs of incubation. **(B)** Kinetics of *in vivo* migration of adoptively transferred FVIII TRuCε or FVIII TRuCε CXCR5 T_conv_ cells to the spleen on days 1, 2, 4, and 7 following adoptive transfer. Mice received i.v. injections of recombinant FVIII on days 0, 3, and 6. Frequencies of mScarlet^+^ cells per total CD4^+^ T cells are quantified by flow cytometry. **(C)** Number of mScarlet^+^ FVIII TRuCε or FVIII TRuCε CXCR5 Tregs per 10^7^ CD4^+^ T cells are quantified from spleens and **(D)** inguinal lymph nodes (ILN) on day 7 post adoptive transfer. Data represents mean±SEM, ****p<0.0001, ∗p < 0.05, ∗∗p < 0.01 using 2-way ANOVA with Tukey’s multiple comparisons analysis for **(A)**, 2-way ANOVA with Sidak’s multiple comparisons analysis for **(B)**, unpaired t test for **(C, D)**.

To validate the functionality of CXCR5 co-expression, we performed an *in vitro* transwell cell migration assay. For this we tested both a 2^nd^ generation FVIII CAR described previously (27), FVIII CAR co-expressing CXCR5, or FVIII TRuCε CXCR5 transduced Tregs. Both engineered CAR and TRuCε Tregs co-expressing CXCR5 showed a superior migratory capacity in response to CXCL13 as compared to cells that did not co-express CXCR5 (**Figure 5A**). Migration was CXCL13 dependent as ~1.4 fold lower migration was observed in response to a CXCL12 gradient (**Figure 5A**). We then tested to see if CXCR5 co-expression improved localization to secondary lymphoid organs by i.v. injecting 4x10^6^ FVIII TRuCε or FVIII TRuCε CXCR5 T_conv_ cells into HA mice. This was followed by 2 IU recombinant FVIII injections administered every 3 days to initiate ADA formation to FVIII. Spleens were harvested on Days 1, 2, 4, and 7 post adoptive transfer, and frequencies of mScarlet^+^ engineered cells were quantified. Frequencies of FVIII TRuCε CXCR5 T_conv_ cells were significantly increased at all timepoints, which was 1.57-1.66 fold higher than FVIII TRuCε T_conv_ (**Figure 5B**, **Supplementary Figure 2**). Next, we confirmed enhanced *in vivo* persistence in both spleens and inguinal lymph nodes (ILN) of HA mice that received 4x10^6^ FVIII TRuCε CXCR5 Tregs. We selected Day 7 as a timepoint to confirm that cells that trafficked into the SLO at earlier timepoints persisted in the organs. FVIII TRuCε CXCR5 Tregs were 1.59 fold higher in the spleen (**Figure 5C**) and 2.72 fold higher in the ILNs (**Figure 5D**) as compared to FVIII TRuCε Tregs, collectively indicating improved *in vivo* persistence of both engineered T_conv_ and Treg in SLOs.

### FVIII TRuCε CXCR5 Tregs suppress ADA formation

**Figure 6:**
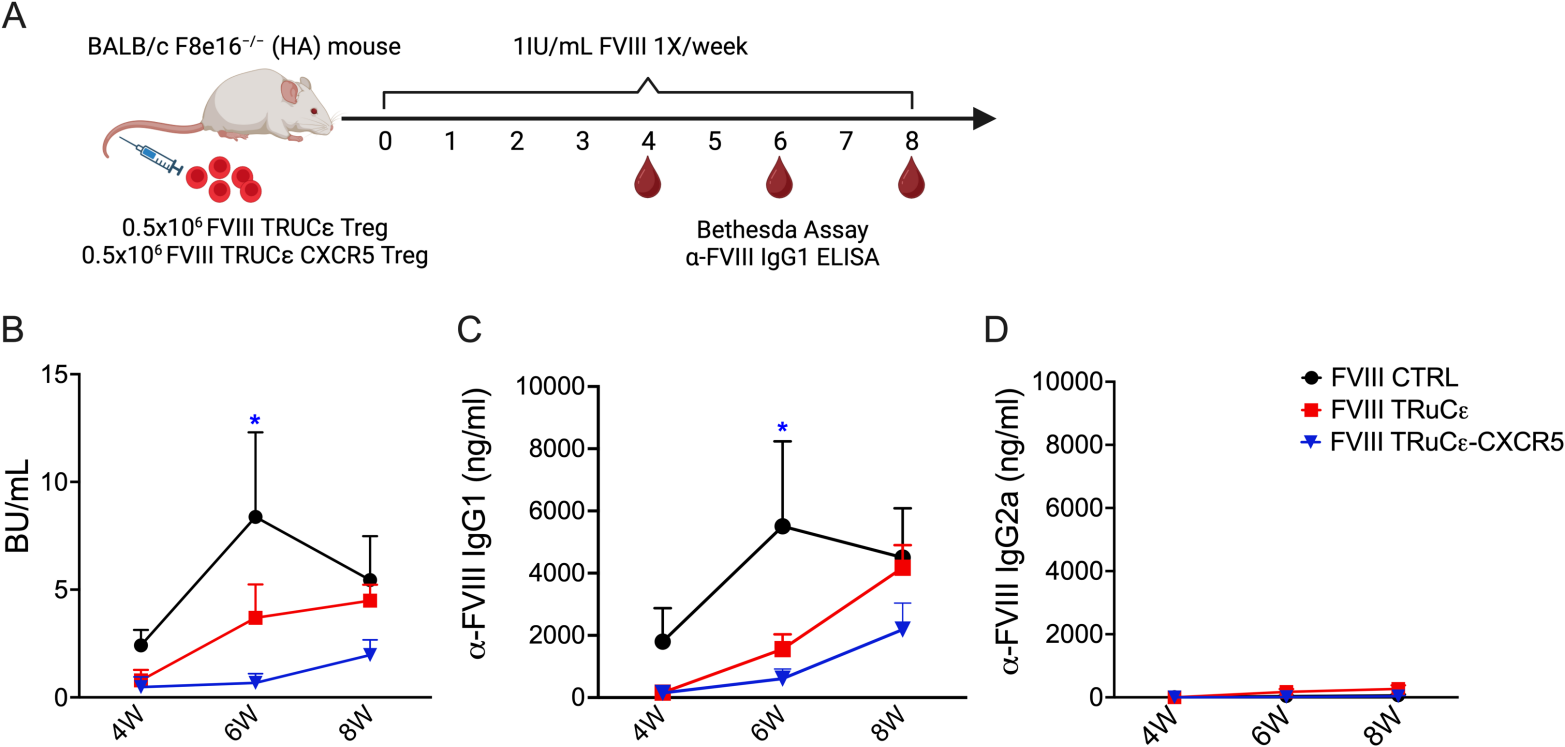
*In vivo* suppression of ADAs to FVIII. **(A)** Schematic representing timeline for assessing *in vivo* prevention of ADA formation engineered Tregs. 0.5 × 10^6^ FVIII TRuCε or FVIII TRuCε CXCR5 Treg were adoptively transferred into BALB/c F8e16^−/−^ (HA) recipient mice (n = 4−10/group). Mice received 8 weekly i.v. injections of 1IU recombinant FVIII, and plasma samples were analyzed after the 4th and 8th injection for **(B)** functional inhibitors by Bethesda assay, **(C)** α-FVIII IgG1 ELISA, and **(D)** α-FVIII IgG2a ELISA. FVIII Control mice received only BDD-FVIII injections without Treg transfer. Data represents mean±SEM. ∗p < 0.05 using 2-way ANOVA with Tukey’s multiple comparisons analysis for **(A)**, 2-way ANOVA with Dunnett’s multiple comparisons analysis for **(B, C)**.

Finally, we investigated whether FVIII TRuCε CXCR5 Tregs maintained a suppressive phenotype *in vivo*. HA recipient mice were i.v. injected with 0.5 × 10^6^ sorted FVIII TRuCε or FVIII TRuCε CXCR5 Tregs followed by 8 weekly i.v. injections of 1 IU recombinant FVIII (**Figure 6A**). Formation of ADAs in plasma was measured longitudinally at 4, 6, and 8 weeks by the Bethesda assay, which measures functional inhibition of clotting in the presence of neutralizing ADAs, and anti-FVIII IgG1 ELISA corresponding to human IgG4, the most prominent subtype in hemophilia A patients with high ADA titers (19, 40). At 4 and 6 weeks, lower inhibitory antibody titers were observed in animals that received FVIII TRuC (0.79±0.48 and 3.69±1.5 BU/mL respectively) and FVIII TRuC CXCR5 Tregs (0.47±0.47 and 0.67±0.42 BU/mL respectively) as compared to animals that received only FVIII weekly injections at 4 and 6 weeks (2.4±0.71 and 8.37±3.9 BU/mL respectively, **Figure 6B**). A reduction in ADA titers was particularly evident at the 6-week timepoint in FVIII TRuCε CXCR5 Treg recipients as compared to FVIII control animals (**Figure 6B**). ADA titers rebounded by 8 weeks, which correlated with low persistence of adoptively transferred Tregs in immunocompetent recipients as observed earlier (27). Anti-FVIII IgG1 antibody levels corroborated these findings (**Figure 6C**). To confirm that antibodies were suppressed and not skewed toward a different sub-type, we performed ELISA for anti-FVIII IgG2a, which is considered a functional equivalent to IgG1 in humans (40, 41). Anti-FVIII IgG2a antibody levels were much lower than IgG1 for all groups (**Figure 6D**), indicating that antibodies were not skewed to a different subtype.

## Discussion

Recent studies on Treg engineering are aimed at enhancing *in vivo* persistence, suppressive capacity, or tissue localization. Some of these strategies include arming CAR-Tregs with cytokines such as IL-2 (42–44), OX40L to improve suppression of antigen presenting cells (44), or enriching for chemokine receptors such as CXCR4 to improve trafficking to the bone marrow (45). In this study, we show using diverse receptor engineered constructs, distinct disease models, and in 2 different species, that co-expressing the CXCR5 chemokine receptor augments *in vivo* trafficking and persistence of engineered Tregs in secondary and tertiary lymphoid organs. We successfully developed human anti-HLA-A2 CAR-Tregs and mouse FVIII TRuCε Tregs that co-express the CXCR5 chemokine receptor. Our study demonstrates that both anti-HLA-A2 CXCR5^+^ CAR-Tregs and FVIII TRuCε CXCR5 Tregs remain immune suppressive while acquiring superior migratory capacity. Our combined studies show that CXCR5 engineered Tregs can effectively traffic to sites of antigen expression, such as transplanted islets, without causing either graft rejection or GvHD, consistent with previous work in NSG mice infused with anti-HLA-A2 CAR-Tregs (29). Similarly, enhanced migration and persistence in the spleen and inguinal lymph nodes aid in the control of ADA formation at the site of immune response initiation against FVIII protein replacement therapy.

These findings are significant as they highlight the safety and potential therapeutic utility of engineered Tregs in transplantation and other settings. Additionally, the similar levels of human CD45^+^ chimerism in the peripheral blood and a comparable distribution in the spleen and graft sites between T_conv_ and Treg groups indicate that the trafficking ability of these cells is not compromised by the modifications. It is currently unknown whether the endogenous TCR impacts the function of engineered Tregs. However, previous studies have provided insights into this question. Notably, experiments involving islet transplantation have shown that anti-HLA-A2 CAR-Tregs, with or without their endogenous TCR, efficiently traffic to the site of antigen expression (29). Further optimizations to extend Treg suppressive capacity could include repeat dosing or additional engineering of cytokine payload design such as IL-2 to improve targeted expansion of engineered Tregs (42, 44).

Distinct from CAR-T_convs_, CAR-Tregs showed a low frequency of engineered cells in the graft at the end of the experiment in mice transplanted with pancreatic islets. As previously demonstrated in several pre-clinical mouse models, engineered Tregs show a limited persistence, up to 3 weeks, once infused *in vivo* (28). Due to the good safety profile of the infused anti-HLA-A2 CAR-Tregs, the experiment was stopped after 30-35 days. Considering the reduced CAR-Treg persistence *in vivo*, the length of the experiment might have affected the detectability of engineered cells in the graft at sacrifice. However, considering the preferential CXCR5 expression in the CAR-Treg group compared to UT Treg, we can also hypothesize CAR undetectability due to CAR down-regulation in the presence of the antigen. Further studies are required to address this question.

In conclusion, our findings support the safety and efficacy of anti-HLA-A2 CXCR5^+^ CAR-Tregs in promoting transplant tolerance without causing overt toxicity or GvHD. We additionally find that FVIII TRuCε CXCR5 Tregs accumulate in secondary lymphoid organs and suppress ADA formation to the therapeutic protein FVIII. These results contribute to the growing body of evidence that Tregs engineered to enhance trafficking and regulatory function hold promise for clinical applications in transplantation and potentially other immune-mediated diseases. Further studies are warranted to explore the full therapeutic potential and mechanistic aspects of engineered Tregs in diverse immunological contexts.

## Materials and methods

### Cloning and lentivirus production

A second generation anti-HLA-A2 CAR construct was generated by cloning the antigen-specific single chain fragment variant (scFv) into a CAR encoding bi-directional lentiviral vector (LV) backbone, containing an NGFR-derived spacer (46), a CD28 transmembrane and co-stimulatory domain and a CD3 zeta endodomain under the control of the human phosphoglycerate kinase (hPGK) promoter. In anti-sense, the human CXCR5 gene was cloned under the control of a minimal cytomegalovirus (mCMV) promoter. LV backbones were kindly provided by L. Naldini’s group. Third generation replication-defective and self-inactivating LV were produced by transient transfection of HEK-293T cells. Briefly, a solution containing the packaging plasmids pMDLg/pRRE (containing HIV-1 gag/pol genes), the pILVV01-Rev plasmid, the envelope plasmid encoding the vesicular stomatitis virus glycoprotein (VSV-G), pAdvantage (that enhances transient protein expression) and plasmids carrying the transgene of interest were transfected in sub-confluent 293T cells using the calcium chloride precipitation method. Supernatants containing lentiviral particles were collected 48 hours later and filtered using a 0.22 μm filter, ultracentrifuged at 8000 rpm for 18 h at 4°C (Beckman Optima XL-100K Ultracentrifuge), aliquoted, and cryopreserved.

### Human PBMC and T cell isolation and expansion

Human buffy coats from de-identified healthy donors were used according to protocols approved by the Institutional Review Board (IRB). Peripheral blood mononuclear cells (PBMCs) were isolated by Ficoll (GE Healthcare, Chicago, IL) density gradient centrifugation. Tregs were enriched using the CD4^+^CD25^+^ human Treg cell isolation kit (Miltenyi Biotec, Auburn, CA), as per the manufacturer’s instructions. For some studies, both CD4^+^CD25^-^ T_conv_ and CD4^+^CD25^+^ Tregs were used. Tregs were expanded and transduced as previously described (28). Briefly, Tregs were stimulated with anti-CD3/CD28 stimulation beads with a Treg: bead ratio = 1:3 (Dynabeads, ThermoFisher) and cultured in rapamycin. At Day +2 after stimulation, activated Tregs were harvested and transduced with a lentiviral vector at a multiplicity of infection (MOI) of 10. After 24 hours, infection was stopped by adding fresh culture medium. Starting from Day +3 and every 3 days 500 IU/ml of IL2 were added to the culture. Starting from Day +14, engineered cells were employed for functional assays. Transduction efficiency was assessed at Day +14 by flow cytometry. CAR expression was identified as the percentage of NGFR^+^ cells. Antibodies utilized for flow cytometry are summarized in **Supplementary Table 1**.

### *In vitro* CAR-Treg suppression assays

Anti-HLA-A2 CXCR5^+^ CAR-Treg suppressive capacity was tested upon polyclonal stimulation. Autologous T_conv_ were co-cultured with either engineered or unmanipulated Tregs in decreasing Treg: T_conv_ ratio (1:1 to 1:16) in the presence of anti-CD3/CD28 stimulation beads (Dynabeads, ThermoFisher) at a bead: T cell ratio = 1:10. To detect proliferating cells, T_conv_ were labeled with a cell trace proliferation dye (VioBlue, ThermoFisher), according to the manufacturer’s instructions. T_conv_ proliferation was assessed after 7 days of culture. Suppression Index (SI) was calculated as 1 – (% of proliferating T_conv_ in each condition/% of proliferating T_conv_ alone).

To test their antigen specific suppression, autologous T_conv_ were transduced to express the anti-NY-ESO-1 TCR and were labeled with VioBlue, according to the manufacturer’s instructions. Anti-NY-ESO-1 TCR T_conv_ were co-cultured with either anti-HLA-A2 CXCR5^+^ CAR- or unmanipulated Tregs in a decreasing Treg: T_conv_ ratio (1:1 to 1:16). To provide a stimulatory signal to anti-NY-ESO-1 TCR T_conv_, HLA-A2^+^ NY-ESO-1^+^ U266 tumor cells were added at a fixed T_conv_: U266 ratio of 2:1. Proliferation of labeled anti-NY-ESO-1 T_conv_ was assessed after 3 days of culture, as previously described (47).

### *In vitro* CAR-Treg migration assay

To assess *in vitro* migration, anti-HLA-A2 CXCR5^+^ CAR- or UT Tregs were cultured in 24 well/plate transwells with 3 μm pores (ThermoFisher) in IMDM medium + 0.1% BSA, with increasing concentrations of hCXCL13. Cells were incubated at 37°C with 5% of CO_2_ and migration was assessed after 4 hours. Migrating cells were collected, counted and their CXCR5 expression was assessed by flow cytometry.

To test the killing activity, HLA-A2^+^ U266 tumor cells were cultured in a 96 well/plate in RPMI 1640 (Lonza) together with Tregs (engineered or unmodified), or with anti-HLA-A2 CAR-T_convs_ at a fixed ratio of 1:5 (T cell: U266). The number of live U266 was assessed after 3 days by flow cytometry. Cells were labeled with DAPI to assess vitality and were counted with flow-count fluorospheres (Beckman Coulter) using the formula provided by the manufacturer.

### Human B cell and peripheral helper T cell isolation

Human HLA-A2^+^ B cells were isolated from healthy donors’ PBMCs through MACS cell separation (B Cell Isolation Kit II, Miltenyi). HLA-A2^+^ peripheral follicular helper T cells were isolated as CD4^+^ CXCR5^+^ elements from healthy donors’ PBMCs by pre-enriching the cells for CD4^+^ T lymphocytes by magnetic cell separation (CD4 T cell isolation kit human, Miltenyi) and further refining the sorting by MACSQuant Tyto Cell Sorter.

### B cell maturation assay

Autologous HLA-A2^+^ B cells and peripheral follicular helper T cells were cultured in a 96 well/plate in RPMI 1640 (Lonza). To induce B and Tfh cell activation and differentiation, cells were stimulated with 5 ug/mL of anti-human IgM F(ab′)2 fragment as previously reported (48), (Sigma-Aldrich) and 2 ug/mL of anti-human CD3 antibody, functional grade (Miltenyi Biotec), respectively. To avoid the unspecific activation of Tregs, after 24 hours the plate was centrifuged, and the supernatant was aspired to remove the anti-CD3 antibody. Engineered or unmodified Tregs were then added and anti-human IgM was replenished to restore the initial concentration. The number of IgG^+^ cells was assessed by flow cytometry 6 days later.

### Islet transplantation

Female or male NSG mice were rendered diabetic by a single intraperitoneal (i.p.) injection of streptozotocin (STZ) at 220 mg/kg and islets were transplanted 72-96 hours later. Blood glucose levels were monitored 2-3 times per week using a glucometer. Only mice with blood glucose levels above 300 mg/dl were used for transplantation. Pancreatic islets from NSG.HLA-A2 transgenic mice (NSG-HLA-A2/HHD, Jackson Laboratories, Bar Harbor, ME) were isolated as previously described (49). A total of 500 mouse islets were transplanted under the kidney capsule. Blood glucose levels of <250mg/dl on two consecutive days were defined as successful islet engraftment. Anti-HLA-A2 CXCR5^+^ CAR-Tregs or T_conv_ cells or UT Tregs were infused intravenously at the dose of 2x10^6 cells/mouse in transplanted mice. All NSG mouse experiments were performed according to San Raffaele Institutional Animal Care and Use Committee approved protocol (IACUC#1019).

### FVIII TRuC cloning and retrovirus production

The FVIII scFv, directed to the C1 and C2 domains of FVIII, was derived from an Epstein-Barr virus (EBV)-transformed B cell clone obtained from a hemophilia A (HA) patient (originally developed by Jacquemin and colleagues, kindly provided to us by Dr. David Scott, Uniformed Services University, Bethesda, MD, USA). The scFv was constructed from the variable heavy (V_H_) and light (V_L_) antibody sequences (Creative Biolabs, Shirley, NJ, USA) and complexed to the N terminus of murine CD3ϵ by a flexible linker (G4S) X3 to generate the FVIII TRuCϵ construct. Murine CXCR5 was co-expressed separated by a P2A sequence (GenScript, Piscataway, NJ, USA). FVIII TRuCϵ CXCR5 was inserted into the pMYs-IRES-mScarlet retroviral backbone. Transfer plasmids were transfected into the PlatE ecotropic retroviral packaging cell line (Cell Biolabs, San Diego, CA) using polyethylenimine (PEI) transfection reagent, and retrovirus containing supernatants were collected after 48 h and passed through a 0.22μM vacuum filter.

### Mouse Treg isolation and retroviral transduction

BALB/c FoxP3 IRES-GFP (Foxp3-GFP) mice were purchased from The Jackson Laboratory (Bar Harbor, ME, USA) and bred in house at Indiana University. Both male and female mice were used as Treg donors for *in vitro* studies, whereas male mice were used as Treg donors for adoptive transfer studies, indicative of the X-linked disorder. CD4^+^CD25^−^ T_conv_ or CD4^+^CD25^+^ Tregs from Foxp3-GFP mice were magnetically enriched using a mouse CD4^+^CD25^+^ Treg isolation kit (Miltenyi Biotec, Auburn, CA, USA), and further purified by four-way purity cell sorting (FACSAria II or FACSAria SORP, BD Biosciences). Cells were pre-activated for 48 h with a 1:1 bead-to-cell ratio using α-CD3/28 mouse microbeads (Dynabeads, Invitrogen, Carlsbad, CA). T_conv_ and Tregs were cultured in Immunocult-XF T cell expansion medium (StemCell Technologies, Vancouver, BC) supplemented with 5% fetal bovine serum (FBS; Atlanta Biologicals, Norcross, GA, USA), 10,000 IU/mL penicillin, 10 mg/mL streptomycin, 1× GlutaMAX-1, 1 mM sodium pyruvate, 10 mM HEPES, 1× nonessential amino acids, and 10 μM 2-mercaptoethanol. Clinical-grade recombinant hIL-2 (Proleukin/aldesleukin; Prometheus Therapeutics and Diagnostics, San Diego, CA) was added at a final concentration of 100 IU/mL for T_conv_ and 1,000 IU/mL for Tregs. Cells were transduced by spinoculation with retrovirus containing supernatants at 1,200 × g for 90 min in non-tissue culture-treated 6-well plates coated with 20 μg/mL retronectin (Takara Bio, Middleton, WI). Transduced cells were *ex vivo* expanded for 3−4 days in the presence of α-CD3/28 microbeads at a 1:1 bead-to-cell ratio. Cells were rested for 4−6 h prior to functional *in vitro* or *in vivo* experiments.

### *In vitro* activation and migration assay

For *in vitro* activation, 1 × 10^6^ untransduced (UT) or FVIII TRuCϵ CXCR5 Tregs were stimulated with 5 IU/mL of recombinant human B domain deleted (BDD) FVIII (Xyntha; Pfizer, New York, NY, USA) or recombinant human FIX (Benefix; Pfizer, New York, NY, USA) for 48hrs, following which they were subject to flow cytometry analysis. A complete table of all the antibodies used is listed in **Supplementary Table 1**.

For *in vitro* migration, FVIII CAR, FVIII CAR CXCR5 and FVIII TRuCϵ CXCR5 Tregs were serum starved overnight and 0.5 × 10^6^ cells/well placed in the upper chamber of 24 well cell culture plates with 5µm pore polycarbonate membrane inserts (Costar Transwell, Corning, NY). Lower chambers contained 600 μL serum-free medium with 1μg/mL of either recombinant mouse CXCL12 or CXCL13 (PeproTech, Rocky Hill, NJ). Serum-free medium with no added chemokine served as a negative control. Cells in the upper chamber were allowed to migrate for 6 h at 37°C and 5% CO_2_. Migrated cells were collected from the lower compartment and quantified on a BD Fortessa flow cytometer.

### ADA formation and analysis of plasma samples in HA mice

BALB/c F8e16^−/−^ hemophilia A (HA) mice were originally provided by Dr. David Lillicrap (Queens University, Ontario, Canada). Animals were housed under specific pathogen-free conditions at Indiana University (Indianapolis, IN) and treated under Institutional Animal Care and Use Committee-approved protocols. Male mice were used for studies involving adoptive transfer or ADA formation. HA mice (n = 5−7) received weekly i.v. administrations of 1 IU B domain deleted (BDD) recombinant FVIII (Xyntha, Pfizer, New York, NY). Mice received 0.5 × 10^6^ FVIII TRuCϵ CXCR5 Tregs 1 day prior to starting FVIII injections. At 4-, 6-, and 8-week time points post-adoptive transfer, ∼200 μL blood was collected from the retroorbital plexus using non-treated capillary tubes into 3.8% sodium citrate, and plasma was analyzed for inhibitor formation by the Bethesda assay (measured on a Diagnostica Stago STart Hemostasis Analyzer; Stago, Parsippany, NJ, USA), anti-FVIII IgG1 and IgG2a ELISA as previously described (50). 1 BU is defined as the reciprocal of the dilution of test plasma at which 50% of FVIII activity is inhibited.

### Statistical analysis

All experiments were independently repeated at least 2 times. Data shown are mean ± SEM. Statistical significance was determined using Mann-Whitney test for non-parametric data and unpaired t test, 1-way or 2-way ANOVA for parametric data, and multiple comparisons were made using Dunnett’s, Tukey’s, Sidak’s, or Brown-Forsythe post-tests as indicated, using GraphPad Prism 10 software (GraphPad, La Jolla, CA, USA). Values at p < 0.05 were deemed significant and indicated as follows: ∗p < 0.05, ∗∗p < 0.01, ∗∗∗p < 0.001, ∗∗∗∗p < 0.0001.

## Data Availability

The original contributions presented in the study are included in the article/supplementary material, further inquiries can be directed to the corresponding author/s.

## Supplementary material

Supplementary Figure 1 Schematic representation of the CAR Treg generation protocol. **(A)** Schematic representation of CAR Treg generation. CD4^+^CD25^+^ Tregs were isolated from peripheral blood mononuclear cells (PBMCs) of healthy donors using magnetic cell sorting and stimulated with anti-CD3/CD28 beads (Treg: bead ratio = 1:3) in the presence of rapamycin. At Day +2, Tregs were transduced with the bi-directional lentiviral vector encoding for a second-generation anti-HLA-A2 CAR construct and the human CXCR5 gene. Starting from Day +3, 500 IU/ml IL-2 was added and replenished every 3 days. At Day +14, beads were detached and employed for functional studies. **(B)** Representative plot of anti-HLA.A2 CAR and hCXCR5 co-localization assessed by flow cytometry. **(C)** Expansion rate of UT and CAR-Tregs at Day +14, expressed as fold increase calculated by dividing the number of cells at Day +14 with the number of cells at day 0. **(D)** Frequencies of FoxP3^+^CD25^+^ and **(E).** FoxP3^+^Helios^+^ CAR Treg and T_convs_. F. Relative FoxP3, Helios, and CD25 expression on CAR Treg and T_convs_. **(G)** Representative plot of CXCR5^+^ cell sorting on Tfh cells (Miltenyi Tyto cell sorter). **(H)** Treg killing capacity against an HLA-A2^+^ U266 cell line. Treg: U266 ratio = 1:5. CAR-T_conv_ were employed as positive control. Killing was assessed after 3 days of culture evaluating the number of live tumor cell by flow cytometry. Results are expressed as Elimination Index (EI) calculated as live tumor cells in the Treg condition/live tumor cells in the CAR-T_conv_ condition. Results are expressed as mean ± SD and N = 3.

Supplementary Figure 2 Representative density plots comparing frequencies of adoptively transferred FVIII TRuCε or FVIII TRuCε CXCR5 T_conv_ cells in spleens of recipient mice on days 2, 4, and 7 following adoptive transfer. Frequencies of mScarlet^+^ cells per total CD4^+^ T cells are quantified by flow cytometry.
