# Supplementary figures and images for: CXCR5 engineered human and murine Tregs for targeted suppression in secondary and tertiary lymphoid organs
Source: Front Immunol. 2025 Jul 1;16:1513009. doi: 10.3389/fimmu.2025.1513009 (PMC12261343; doi:10.3389/fimmu.2025.1513009)

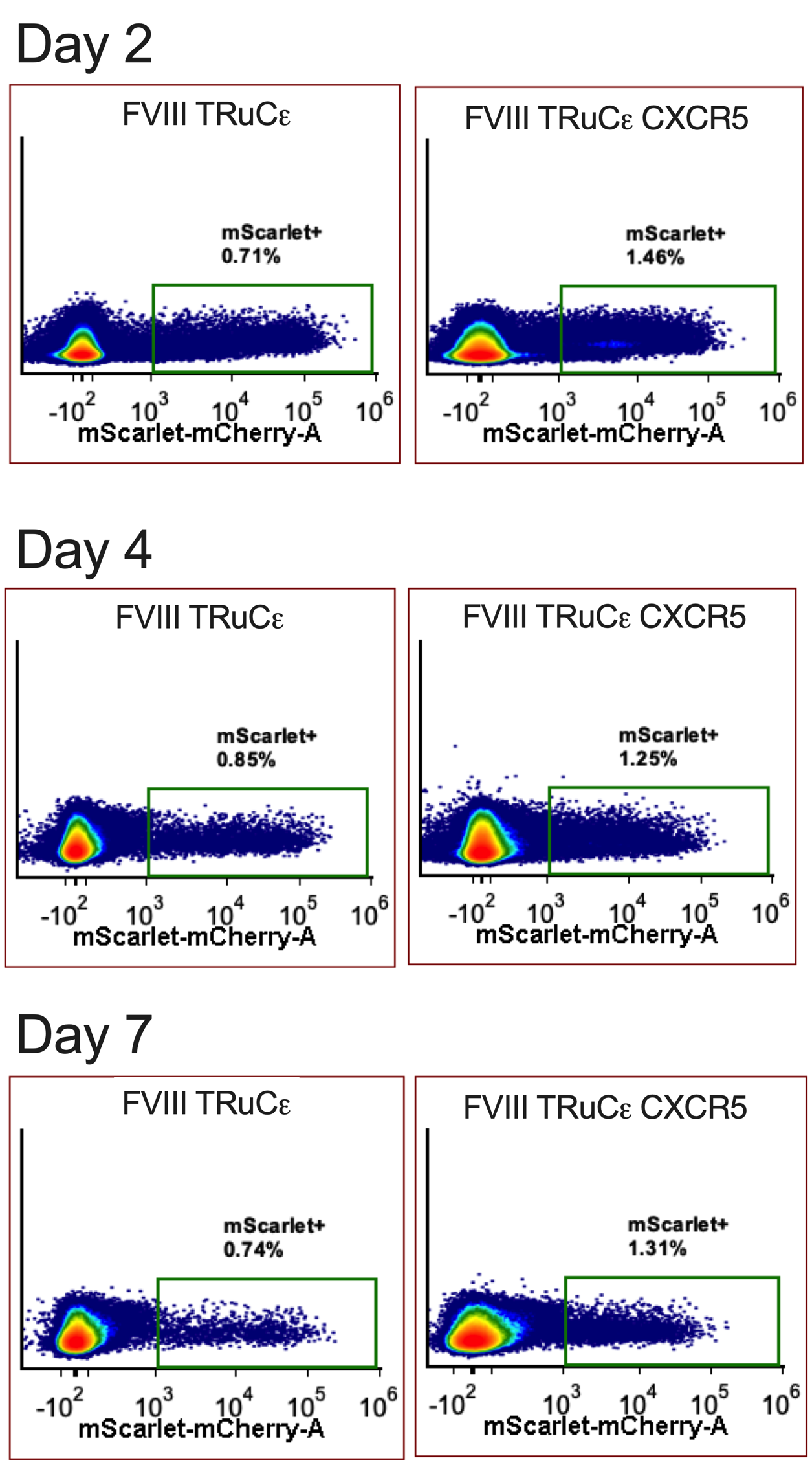

Supplement: Supplementary Figure 1 — Schematic representation of the CAR Treg generation protocol. (A) Schematic representation of CAR Treg generation. CD4+CD25+ Tregs were isolated from peripheral blood mononuclear cells (PBMCs) of healthy donors using magnetic cell sorting and stimulated with anti-CD3/CD28 beads (Treg: bead ratio = 1:3) in the presence of rapamycin. At Day +2, Tregs were transduced with the bi-directional lentiviral vector encoding for a second-generation anti-HLA-A2 CAR construct and the human CXCR5 gene. Starting from Day +3, 500 IU/ml IL-2 was added and replenished every 3 days. At Day +14, beads were detached and employed for functional studies. (B) Representative plot of anti-HLA.A2 CAR and hCXCR5 co-localization assessed by flow cytometry. (C) Expansion rate of UT and CAR-Tregs at Day +14, expressed as fold increase calculated by dividing the number of cells at Day +14 with the number of cells at day 0. (D) Frequencies of FoxP3+CD25+ and (E). FoxP3+Helios+ CAR Treg and Tconvs. F. Relative FoxP3, Helios, and CD25 expression on CAR Treg and Tconvs. (G) Representative plot of CXCR5+ cell sorting on Tfh cells (Miltenyi Tyto cell sorter). (H) Treg killing capacity against an HLA-A2+ U266 cell line. Treg: U266 ratio = 1:5. CAR-Tconv were employed as positive control. Killing was assessed after 3 days of culture evaluating the number of live tumor cell by flow cytometry. Results are expressed as Elimination Index (EI) calculated as live tumor cells in the Treg condition/live tumor cells in the CAR-Tconv condition. Results are expressed as mean ± SD and N = 3. [file Image1.tiff]

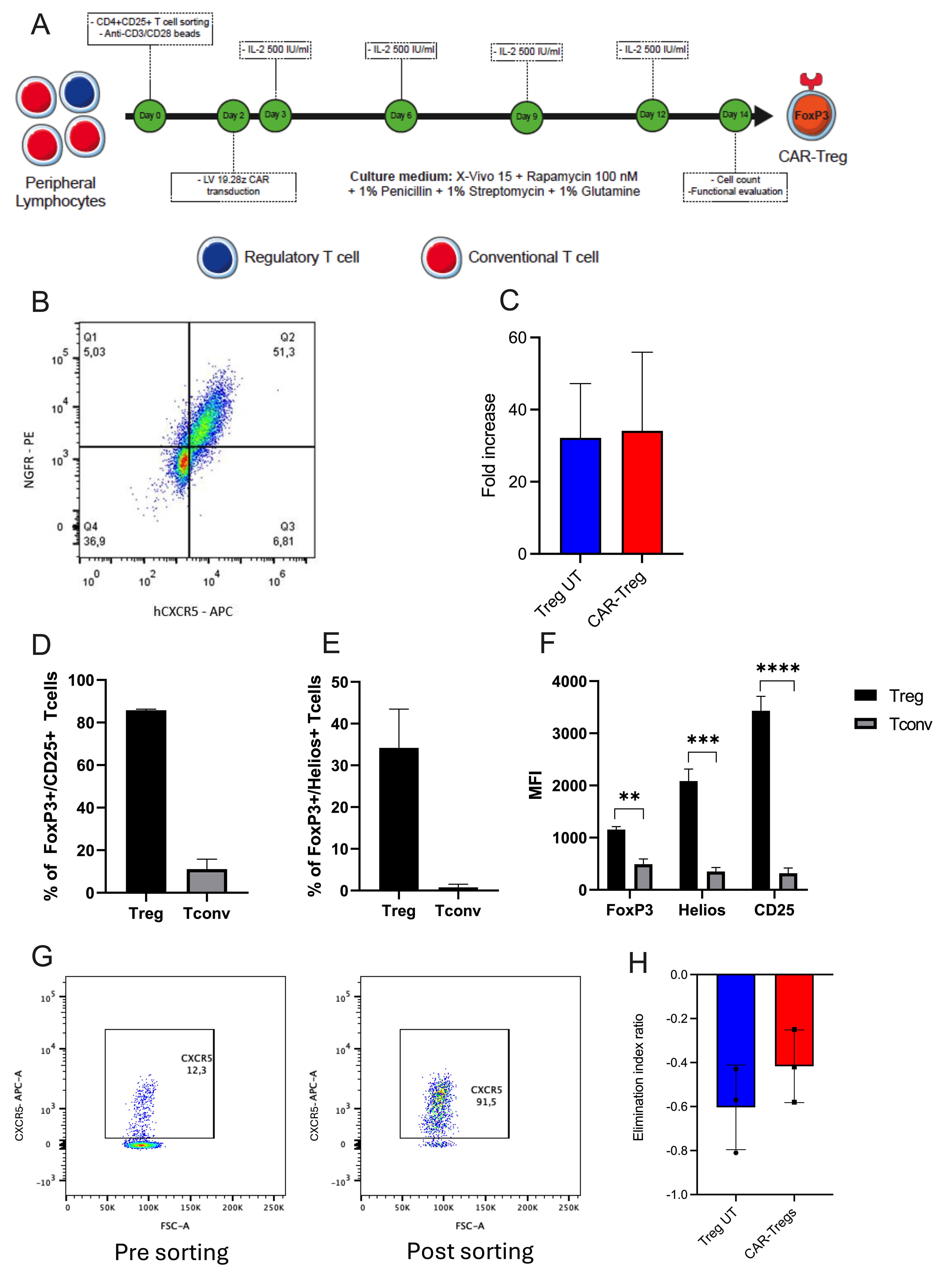

Supplement: Supplementary Figure 2 — Representative density plots comparing frequencies of adoptively transferred FVIII TRuCε or FVIII TRuCε CXCR5 Tconv cells in spleens of recipient mice on days 2, 4, and 7 following adoptive transfer. Frequencies of mScarlet+ cells per total CD4+ T cells are quantified by flow cytometry. [file Image2.tiff]
