# Supplementary material for: CXCR5 engineered human and murine Tregs for targeted suppression in secondary and tertiary lymphoid organs
Source: Front Immunol. 2025 Jul 1;16:1513009. doi: 10.3389/fimmu.2025.1513009 (PMC12261343; doi:10.3389/fimmu.2025.1513009)
Supplement: Supplementary file 3 [file Table1.docx]

**Supplementary Table 1. Antibodies for flow cytometry analysis**

| **Antibody** | **Tag** | **Catalog #** | **Company** |
| --- | --- | --- | --- |
| CXCR5 | BV421 | 145511 | Biolegend |
| CXCR5 | BV785 | 145523 | Biolegend |
| hCXCR5 | AF647 | 558113 | BD Bioscience |
| CD69 | APC | 104513 | Biolegend |
| Helios | APC | 137221 | Biolegend |
| CD25 | BV605 | 102035 | Biolegend |
| CD28 | PerCP/Cy5.5 | 102113 | Biolegend |
| Ki67 | BV605 | 652413 | Biolegend |
| PDL1 | APC | 124311 | Biolegend |
| PD1 | APCeFluor780 | 135239 | Biolegend |
| LAP | PerCP/Cy5.5 | 141409 | Biolegend |
| ICOS | BV421 | 117429 | Biolegend |
| CTLA4 | BV421 | 106311 | Biolegend |
| LAG3 | BV421 | 125221 | Biolegend |
| TIM3 | PerCP/Cy5.5 | 134011 | Biolegend |
| FoxP3 | eFluor660 | 50-5773-82 | eBioscience |
| FoxP3 | Pe-Cy5 | 15477641 | eBioscience |
| IRF4 | PerCP/Cy5.5 | 646415 | Biolegend |
| GATA3 | BV421 | 653813 | Biolegend |
| TBet | BV711 | 644819 | Biolegend |
| CD3 | eFluor506 | 69003742 | Invitrogen |
| CD4 | PE-Vio615 | 130-113-226 | Miltenyi |
| CD25 | PE-Vio770 | 130-116-205 | Miltenyi |
| CD127 | APC-Vio770 | 130-113-416 | Miltenyi |
| CD271 | PE | 560927 | BD Bioscience |
| Vβ 13.1 | FITC | 11-5792-41 | eBioscience |
| HLA-A2 | PE | 12-9876-42 | eBioscience |
| IgG Fc | AF488 | A55747 | Invitrogen |
